# Structural, Optical, and Magnetic Properties of NiMoO_4_ Nanorods Prepared by Microwave Sintering

**DOI:** 10.1155/2015/315084

**Published:** 2015-02-23

**Authors:** Ana P. de Moura, Larissa H. de Oliveira, Ieda L. V. Rosa, Camila S. Xavier, Paulo N. Lisboa-Filho, Máximo S. Li, Felipe A. La Porta, Elson Longo, José A. Varela

**Affiliations:** ^1^Instituto de Química, UNESP, 14800-900 Araraquara, SP, Brazil; ^2^Departamento de Química, UFSCar, 13565-905 São Carlos, SP, Brazil; ^3^Departamento de Física, UNESP, 13565-905 Bauru, SP, Brazil; ^4^Departamento de Física, USP, 13560-970 São Carlos, SP, Brazil

## Abstract

We report on the structural, optical, and magnetic properties of *α*,*β*-NiMoO_4_ nanorods synthesized by annealing the NiMoO_4_:*n*H_2_O precursor at 600°C for 10 minutes in a domestic microwave. The crystalline structure properties of *α*,*β*-NiMoO_4_ were investigated using X-ray diffraction (XRD), Fourier transform infrared (FTIR), and Raman (FT-Raman) spectroscopies. The particle morphologies and size distributions were identified by field emission microscopy (FE-SEM). Experimental data were obtained by magnetization measurements for different applied magnetic fields. Optical properties were analyzed by ultraviolet-visible (UV-vis) and photoluminescence (PL) measurements. Our results revealed that the oxygen atoms occupy different positions and are very disturbed in the lattice and exhibit a particular characteristic related to differences in the length of the chemical bonds (Ni-O and Mo-O) of the cluster structure or defect densities in the crystalline *α*,*β*-NiMoO_4_ nanorods, which are the key to a deeper understanding of the exploitable physical and chemical properties in this study.

## 1. Introduction

Over the past few decades, morphology as well as size control of crystalline materials is the key requirement for their industrial exploitation with unprecedented capabilities in emerging technologies [1–4]. In this context, nickel molybdates (NiMoO_4_) have received much attention, due to their remarkable record of widespread applications in different fields of materials science, such as photocatalysis, phosphors, light-emitting diodes, optical fibers, humidity sensors, scintillators, and magnetic properties [5]. Under atmospheric pressure, three compounds of NiMoO_4_ are known: the low temperature *α*-phase, the high temperature *β*-isomorph, and the hydrate and NiMoO_4_:*n*H_2_O, respectively [6–10]. Both *α*-NiMoO_4_ and *β*-NiMoO_4_ are often obtained by heating their nickel molybdate hydrates NiMoO_4_:*n*H_2_O that is utilized as a precursor [11]. On the other hand, the synthesis of the pure *β*-NiMoO_4_ phase is usually generated by heating the *α*-NiMoO_4_ at temperature above 760°C; however, the *β*-NiMoO_4_ phase is stable only above 180°C due that on cooling below at this temperature transforms again into the *α*-NiMoO_4_ phase, which is more stable under these conditions [12–15]. These studies have examined the catalytic properties of both phases and in all these cases the *β*-NiMoO_4_ phase showed a higher efficiency in these tests. However, very little has been reported on the optical properties of these systems.

It has been known that preparation and conditions methods become essential to control the obtention of semiconductor nanomaterials with the tunable physical and chemical properties, which are very important in a wide range of applications in nanotechnology [16–18]. Recently, the domestic microwave oven has been successfully employed to obtain many ceramic materials with improved quality and size distributions of the nanomaterials [19–22]. Advantages such as rapid heating, selective material coupling, and enhanced reaction kinetics make the microwave process an attractive route for these materials' synthesis [23–27], showing significant advantages against conventional sintering procedures [28–30]. Recently, Oghbaei and Mirzaee [30] reported a complete review on the subject.

Therefore, in this work, we report the correlation among optical and magnetic properties of the *α*,*β*-NiMoO_4_ nanorods synthesized by annealing the NiMoO_4_:*n*H_2_O precursor at 600°C for 10 minutes in a domestic microwave. Moreover, these nanorods were analyzed by XRD, FTIR, FT-Raman, FE-SEM, UV-vis, and PL, and magnetic behavior was also studied. In this context, the structural and electronic order-disorder effects influence physical properties as it will be shown here. In addition, the effects, as well as the influence, of microwave heating by using the synthetic process are reported.

## 2. Materials and Methods

### 2.1. Synthesis of *α*,*β*-NiMoO_4_ Powders

**Figure 1:**
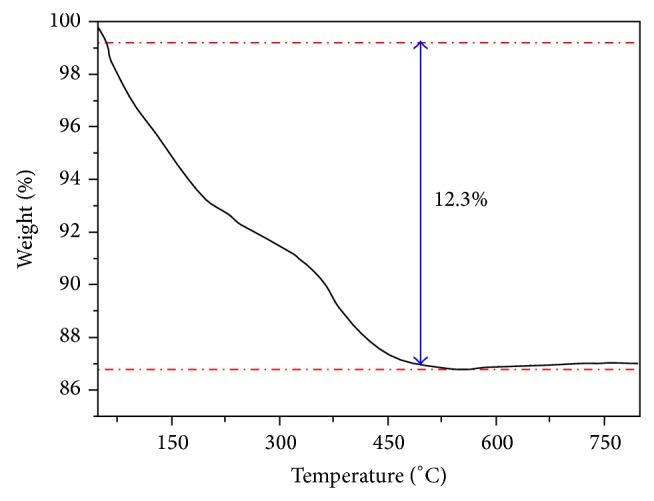
TGA curve of the precursor NiMoO_4_·*n*H_2_O.

In a typical procedure, 2 mmol of sodium molybdate solution was dissolved in 50 mL of distilled water. Afterwards, 2 mmol of nickel nitrate hexahydrate was dissolved in 50 mL of deionized water, which was slowly added into the sodium molybdate solution under magnetic stirring giving rise to a homogeneous solution (pH = 6). The reactional mixture was put under magnetic stirring during 30 minutes. Then, the obtained precipitate was water washed for several times, and the powder of green-yellow color was dried at 60°C for 12 hours under air atmosphere in a conventional furnace. The obtained precursor was investigated using thermal analysis.  Figure 1 shows the TGA curve for the thermal decomposition of the precursor. TGA curves of the NiMoO_4_·*n*H_2_O samples with the temperature ranging from 30 to 800°C show that a mass losses at about 480°C was 12.3% in the net weight, mainly ascribed to the loss of water content in the NiMoO_4_·*n*H_2_O. Therefore we choose 600°C as the heating temperature to obtain the *α*,*β*-NiMoO_4_ nanorods. The *α*,*β*-NiMoO_4_ yellow-like powders were obtained from thermal decomposition of the precursor powders in ceramic crucibles and heated in a microwave sintering furnace at 600°C for 10 minutes.

### 2.2. Characterizations

The powders were characterized by X-ray diffraction (XRD) using a Rigaku-DMax 2500 PC, Japan, with Cu K*α* radiation (*λ* = 1.540598 Å) in the 2*θ* range from 10° to 75° using a scanning rate of 0.02°/min. The phase analysis by the Rietveld and Le Bail method [31, 32] was carried out using the General Structure Analysis System (GSAS) software [33]. FT-IR spectroscopies were performed in the range from 400 to 4000 cm^−1^, using a Bruker-Equinox 55 (Germany) spectrometer in transmittance mode. FT-Raman spectroscopy was recorded with a Bruker-RFS 100 (Germany). The spectra were obtained using a 1064 nm line of Nd:YAG laser, keeping its maximum output power at 110 mW.

The morphologies of the samples were verified using a field emission gun scanning electron microscopy (Jeol JSM 6330F). UV-vis spectra were taken using Cary 5G (Varian, USA) equipment in the diffuse reflection mode. The thermal decomposition of the precursor powders was studied by thermogravimetric analysis (TGA/DTA) on a TGA2050 thermal analysis device (American TA Corporation). TGA determination was carried out in air at a heating rate of 20°C min^−1^ in the range from room temperature to 900°C.

The PL measurements were taken in a Thermal Jarrel-Ash Monospec 27 monochromator and a Hamamatsu R446 photomultiplier. The 350.7 nm exciting wavelength of a krypton ion laser (Coherent Innova) was used with the nominal output power of the laser power kept at 200 mW. All the measurements were taken at room temperature. Magnetization versus an applied field in a zero field cooled (ZFC) and field cooled (FC) measurements was performed using a Quantum Design Magnetic Properties Measurement System (MPMS) XL-5 Superconducting Quantum Interference Device.

## 3. Results and Discussion

**Figure 2:**
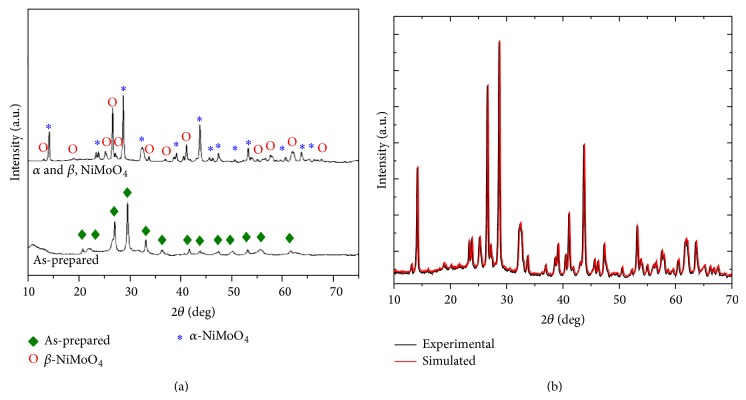
(a) XRD patterns of the as-prepared powder and the powders heated at 600°C for 10 minutes in a microwave oven together with (b) structural refinements plot for the *α*,*β*-NiMoO_4_ nanostructures.

The crystallinity and crystal structures of the precursor and calcined products (*α*- and *β*-NiMoO_4_) were examined by X-ray diffraction (XRD) as shown in Figure 2(a). The precursor composition is mainly associated with the hydrate precursor. This conclusion was possible, since the diffractograms present the characteristic peaks of this matrix according to JCPDS data file number 13-0128 [34]. In the XRD pattern of the precursor calcined all the reflectance peaks can be perfectly indexed to the mixture of the *α*-NiMoO_4_ and *β*-NiMoO_4_ phases that it was possible to identify the presence of intense well defined and sharp diffraction peaks, which are characteristics of solids structurally ordered in a long-range, according to the JCPDS data file numbers 33-948 and 12-348, respectively. Under these conditions we can see that the sample is richer in the *α*-NiMoO_4_ phase. This conclusion is in line with previous work of other groups [34–37].

From the structural point of view, *α*-NiMoO_4_ and *β*-NiMoO_4_ phases have a monoclinic crystal structure (group space C12/m1) and the most important differences between both phases are different coordination for the molybdenum ions in the crystal structure, being octahedral cluster, [MoO_6_], for the *α*-NiMoO_4_ and tetrahedral one, [MoO_4_], for the *β*-NiMoO_4_ powder [30]. In order to analyze and understand whether there are differences in the structural arrangements of both phases in the sample calcined at 600°C for 10 minutes in a domestic microwave, the Rietveld and Le Bail refinement method was employed (see Figure 2(b)). In particular, the Rietveld method is generally restricted to crystalline phases for which structures are well known allowing the quantification of the phase mixtures [38]. However, for the *β*-NiMoO_4_ phase these parameters are not very well known. In this context, the Le Bail method is very similar to the Rietveld method, except that in this method there is no need to make the crystal structure refinement, and allowing an adjustment that can be obtained by the integrated intensity and the positions of all peaks in the XRD profile [32, 39], and this strategy was used here for the structural determination of the *β*-NiMoO_4_ phase. In this respect, during the XRD refinements the structural parameters such as scale factor, background with exponential shift, microstructure, crystal structure, shift lattice constants, profile half-width parameters (*u*, *v*, *w*), lattice parameters, texture, factor occupancy, and atomic site occupancies were optimized using the GSAS program [33]. The XRD refinement results of the unit cell parameters are *a* = 9.602 Å, *b* = 8.769 Å, *c* = 7.665 Å, and *β* = 114.24° for *α*-NiMoO_4_, while *a* = 10.094 Å, *b* = 9.203 Å, *c* = 6.996 Å, and *β* = 107.17° for *β*-NiMoO_4_ phases, respectively. Clearly, the difference between the measured and calculated patterns is considered a way to verify the success of the refinement method, as shown in Figure 2(b); however, it is necessary to check values of the fitting parameters for greater control of these results. In general, the criteria depend on the type of structure, in general, are recommended low values of the Rw (<10%) and *χ* ^2^ (<2) more reliable are the results of the refinement [1]. As can be observed in Figure 2(b), the quality of the XRD refinement was assessed by the values of the fitting parameters (*R*_WP_ = 4.3%, *R*_Bragg_ = 0.9%, and *χ* ^2^ = 1.17) indicating good agreement between refined and observed XRD patterns for the sample calcined at 600°C for 10 minutes in a domestic microwave, and it was noted that the refined parameters are very close to those published in the literature by Haetge et al. [35]. However, some variations in the atomic positions related to oxygen atoms were observed while the nickel and molybdenum atoms remain fixed in their positions within the framework. These results indicate the existence of local structural distortions on the [NiO_8_], [MoO_4_], and [MoO_6_] clusters of *α*,*β*-NiMoO_4_ nanorods synthesized by annealing the NiMoO_4_:*n*H_2_O precursor at 600°C for 10 minutes in a domestic microwave. In this case, the employed strategy provides information on unit cell parameters, so it can not be used for quantification of the phases present in this sample.

**Figure 3:**
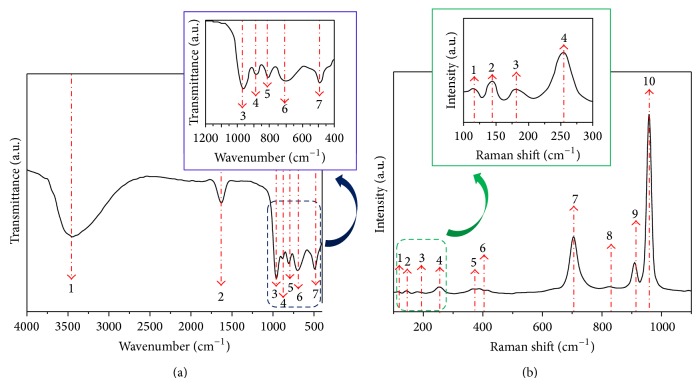
(a) FTIR and (b) FT-Raman spectra of the *α*,*β*-NiMoO_4_ nanorods.

Figures 3(a) and 3(b) illustrate the FTIR and FT-Raman spectra for the *α*,*β*-NiMoO_4_ powders synthesized via the precursor decomposition. According to the FTIR spectra shown in Figure 3(a), the presence of large bands was observed at 3470 cm^−1^ and 1622 cm^−1^, which could be associated with the stretching and flexing modes of the O-H linkages from the water molecules adsorbed in the sample surfaces. The bands at 962 and 882 cm^−1^ can be assigned to the symmetric and antisymmetric stretching of the Mo=O linkage and the band at 492 cm^−1^ could be associated with torsions of the Mo-O-Mo attachment. The bands at 808 and 706 cm^−1^, however, are assigned to the vibrations of the Mo-O-Ni (see Figure 3(a)).  Figure 3(b) shows the FT-Raman spectra of the *α*,*β*-NiMoO_4_ powders indicating that the sample presents structural organization at short range. The results show that the band located at 952 cm^−1^ is associated with the symmetric stretching mode of Mo-O linkage. The bands at 900 and 826 cm^−1^ are due to the asymmetric stretching modes of the oxygen in O-Mo-O link. The bands observed at 380 cm^−1^ and 361 cm^−1^ are related to the bending modes of asymmetric and symmetric O-Mo-O. The band located at 733 cm^−1^ is due to the symmetric stretch of the bond Ni-Mo-O. There is also a band at around 261 cm^−1^ related to deformation modes of Mo-O-Mo linkage. Our results are in very good agreement with other published studies [36, 37, 40, 41].

**Figure 4:**
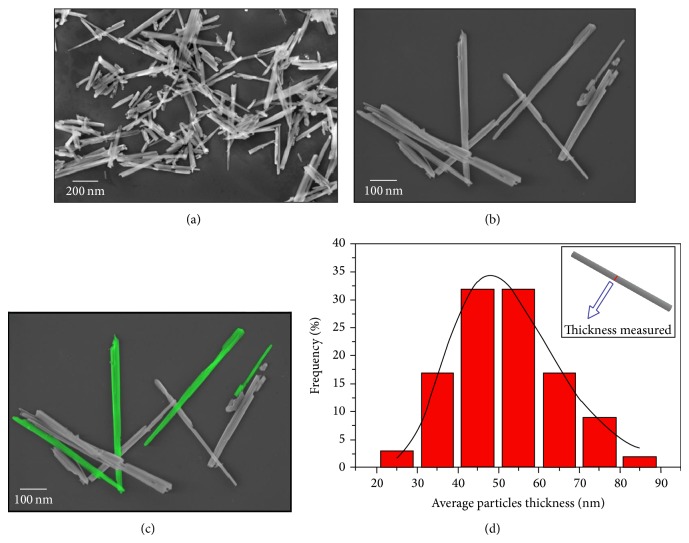
FE-SEM micrograph (a, b, and c) and (d) average distributions of the particle width for *α* and *β*-NiMoO_4_ nanostructures.

Representative FE-SEM micrographs were used to study the particle morphologies and size distributions of *α*,*β*-NiMoO_4_ powders (see Figures 4(a)–4(d)). In particular, FE-SEM images show that the synthesis route formed *α*,*β*-NiMoO_4_ nanorods shape, which corresponds to a polydispersed sample (see Figures 4(a)–4(c)).  Figure 4(d) shows the average distribution of the particles width for *α*,*β*-NiMoO_4_ nanorods. FE-SEM micrographs allowed estimating the average distribution of the particle size for *α*- and *β*-NiMoO_4_ powders through the counting of around 100 particles.  Figure 4(d) shows the average distribution of the particle width in the range from 25 to 85 nm for *α*,*β*-NiMoO_4_ powders. In this figure, 93% of the particles presented an average width from 35 to 65 nm. The diameters of the nanorods were determined as 100–300 nm and the lengths as 1-2 *μ*m.

**Figure 5:**
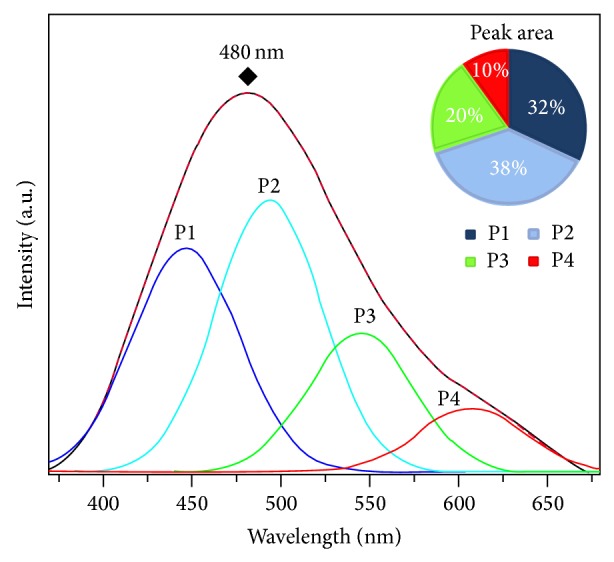
PL spectrum at room temperature and its deconvolution spectra of the *α*,*β*-NiMoO_4_ nanorods.

Owing the higher surface-to-volume ratio in nanoparticles perform quite differently from the corresponding bulk material [9, 42–44]. Figures 5(a) and 5(b) show the UV-vis and PL spectra of the *α*,*β*-NiMoO_4_ nanorods. UV-vis diffuse reflectance was used to determine the optical band gap energy of *α*,*β*-NiMoO_4_ nanorods (for more details on this methodology see [17]). For our sample, the direct optical band gap presented a value of ~2.15 eV, which is in good agreement with values reported in the literature 2.3 eV [45]. The decrease in the band gap value can be attributed to defects and local bond distortion as well as intrinsic surface states and interfaces which yield localized electronic levels within the forbidden band gap [17].

PL emission is considered a powerful tool to obtain information on the electronic structure and degree of structural organization at medium range of the materials [46–49]. The PL spectrum of the *α*,*β*-NiMoO_4_ nanostructures shows a broad band covering a large part of the visible spectrum with a maximum situated at 480 nm (blue emission), when excited by a 350.7 nm laser line (see Figure 5). This PL profile suggests an emission mechanism characterized by the participation of several energy levels or light emission centers able to trap electrons within the band gap. To a better understanding of the properties of PL and its dependence on the structural order-disorder of the lattice, the PL curves were analyzed by the PEAKFIT program [50]. The deconvolution results showed that the PL spectrum was better adjusted by four components (P1 – 446 nm, P2 – 493 nm, P3 – 544 nm, and P4 – 606 nm peak center), and each color represents a different type of electronic transition linked to a specific structural arrangement. The emission band profile is typical of a multiphonon process: that is, a system where relaxation occurs by several paths involving the participation of numerous states within the band gap of the material [46–49].

In previous studies [25, 46–49] we reported that physical behavior for many molybdates compounds having the formula AMoO_4_, where A = Ba, Ca, Pb, Co, and Sr, is explained by a model based on defects or distortions in the lattice, that induce to a symmetry break, process in the crystal and favors to the appearance of new intermediate levels (deep and shallow defects) within the band gap. In this case, the charge gradient between the clusters generates a polarization in the lattice of *α*,*β*-NiMoO_4_ nanorods, can lead to the formation types of distortions on [O-Ni-O] and [O-Mo-O] bonds, and consequently promotes different levels of distortions on the [NiO_8_], [MoO_4_], and [MoO_6_] clusters. These studies have shown that ordered-disordered effects in nano- or microparticles have two types of coordination for Ni, Mo, or O atoms into the lattice, and this phenomenon can be related to the local structure at short, medium, and long range distances and favors the formation of complex cluster vacancies that arise from fast crystallization during the initial nucleation process. Based on these structural and electronic order-disorder effects, for the *α*,*β*-NiMoO_4_ nanorods, the distortion between these complex clusters causes a polarization and/or difference in charge density in the local structure [25, 46–49], which is able to promote a charge transfer from the ${\left[\text{Ni}{\text{O}}_{8}\right]}_{d}^{x}\overset{{\text{e}}^{\prime }}{\to }{\left[\text{Ni}{\text{O}}_{8}\right]}_{o}^{x}$, ${\left[\text{Mo}{\text{O}}_{4}\right]}_{d}^{x}\overset{{\text{e}}^{\prime }}{\to }{\left[\text{Mo}{\text{O}}_{4}\right]}_{o}^{x}$, and ${\left[\text{Mo}{\text{O}}_{6}\right]}_{d}^{x}\overset{{\text{e}}^{\prime }}{\to }{\left[\text{Mo}{\text{O}}_{6}\right]}_{o}^{x}$ complex clusters (*o* = order and *d* = disorder). In particular, the cluster-to-cluster charge transfer (CCCT) process are a natural consequence due to the presence of the structural defects, which essentially is characterized by excitations involving electronic transitions from one cluster to another cluster [49] and are strongly dependent on the formation and recombination of all complex clusters present in the *α*,*β*-NiMoO_4_ crystal was considered and is also represented by Kröger–Vink notation [51] by means of clusters notations in

(1) $$\begin{array}{c} {\left[\text{Ni}{\text{O}}_{8}\right]}_{o}^{x}+{\left[\text{Ni}{\text{O}}_{8}\right]}_{d}^{x}\;⟶{\left[\text{Ni}{\text{O}}_{8}\right]}_{o}^{\prime }+{\left[\text{Ni}{\text{O}}_{8}\right]}_{d}^{•} \end{array}$$

(2) $$\begin{array}{c} {\left[\text{Mo}{\text{O}}_{4}\right]}_{o}^{x}+{\left[\text{Mo}{\text{O}}_{4}\right]}_{d}^{x}\;⟶{\left[\text{Mo}{\text{O}}_{4}\right]}_{o}^{\prime }+{\left[\text{Mo}{\text{O}}_{4}\right]}_{d}^{•} \end{array}$$

(3) $$\begin{array}{c} {\left[\text{Mo}{\text{O}}_{6}\right]}_{o}^{x}+{\left[\text{Mo}{\text{O}}_{6}\right]}_{d}^{x}⟶{\left[\text{Mo}{\text{O}}_{6}\right]}_{o}^{\prime }+{\left[\text{Mo}{\text{O}}_{6}\right]}_{d}^{•} \end{array}$$

**Figure 6:**
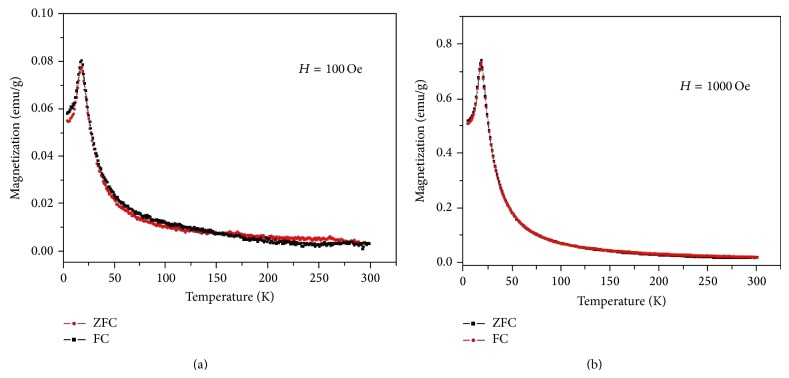
Temperature dependence of the ZFC and FC magnetizations for *α*,*β*-NiMoO_4_ nanorods measured under 100 Oe (a) and 1000 Oe (b) applied fields.

It is assumed that charge redistribution may lead to electron-hole recombination of localized excitons that result in PL behavior for the *α*,*β*-NiMoO_4_ nanorods. Therefore, the structural and electronic reconstructions of all possible combinations of complex clusters belonging to a specific crystal are essential for the deeper understanding of the CCCT process and its influences on the PL phenomenon at the atomic level [49]. In addition, in this study we also investigated the magnetic properties for the *α*,*β*-NiMoO_4_ nanorods. Magnetization, as a function of temperature in a range of 2–300 K for different applied magnetic fields, is shown in Figure 6.

In the “zero-field-cooled” (ZFC) run, the sample was cooled from 300 to 2 K in the absence of an external applied magnetic field. Then, magnetic field was applied and the magnetization was measured as a function of the temperature in the warming process. Following the ZFC run, in the “field-cooled” (FC) run, the sample was then cooled from 300 to 2 K in the presence of an external magnetic field. For both ones, a reversible antiferromagnetic- (AF-) paramagnetic (PM) transition was observed, with an increasing of the magnitude of the magnetization as the magnetic field is increased. The *α*,*β*-NiMoO_4_ nanorods present Nèel temperature (*T*_*N*_) close to 18.5 K (*H* = 100 Oe) and 17.8 K (*H* = 1 kOe) in accordance with the literature [52]. Based on our results, we suggest that the structural and electronic order-disorder effects may contribute to the improvement of PL and magnetic properties of *α*,*β*-NiMoO_4_ nanorods and are mainly associated with the disorder in the medium-range distance created during the processing of these materials.

## 4. Conclusions

In summary, *α*,*β*-NiMoO_4_ nanorods were synthesized by annealing the NiMoO_4_:*n*H_2_O precursor at 600°C for 10 minutes in a domestic microwave. The XRD patterns, FT-Raman, and FTIR spectrum revealed that the nanorods obtained are crystalline structures formed by the *α*-NiMoO_4_ and *β*-NiMoO_4_ phases. UV-vis absorption spectroscopy revealed a characteristic optical band gap of 2.2 eV, which is associated with the difference of energy between the valence and conduction bands. PL emission at room temperature was verified at 480 nm (blue emission), which can be attributed to the participation of several energy levels or light emission centers able to trap electrons within the band gap. For both applied magnetic fields a reversible antiferromagnetic-paramagnetic transition was observed.
